# Markers of Pulmonary Oxygen Toxicity in Hyperbaric Oxygen Therapy Using Exhaled Breath Analysis

**DOI:** 10.3389/fphys.2019.00475

**Published:** 2019-04-24

**Authors:** T. T. Wingelaar, P. Brinkman, P. J. A. M. van Ooij, R. Hoencamp, A. H. Maitland-van der Zee, M. W. Hollmann, R. A. van Hulst

**Affiliations:** ^1^Diving Medical Centre, Royal Netherlands Navy, Den Helder, Netherlands; ^2^Department of Anaesthesiology, Amsterdam UMC, University of Amsterdam, Amsterdam, Netherlands; ^3^Department of Pulmonology, Amsterdam UMC, University of Amsterdam, Amsterdam, Netherlands; ^4^Department of Surgery, Alrijne Hospital Leiderdorp, Leiderdorp, Netherlands; ^5^Defense Healthcare Organisation, Ministry of Defence, Utrecht, Netherlands; ^6^Leiden University Medical Center, Leiden, Netherlands

**Keywords:** hyperbaric oxygen therapy, pulmonary oxygen toxicity, exhaled breath analysis, gas chromatography-mass spectrometry, volatile organic compounds

## Abstract

**Introduction:**

Although hyperbaric oxygen therapy (HBOT) has beneficial effects, some patients experience fatigue and pulmonary complaints after several sessions. The current limits of hyperbaric oxygen exposure to prevent pulmonary oxygen toxicity (POT) are based on pulmonary function tests (PFT), but the limitations of PFT are recognized worldwide. However, no newer modalities to detect POT have been established. Exhaled breath analysis in divers have shown volatile organic compounds (VOCs) of inflammation and methyl alkanes. This study hypothesized that similar VOCs might be detected after HBOT.

**Methods:**

Ten healthy volunteers of the Royal Netherlands Navy underwent six HBOT sessions (95 min at 253 kPa, including three 5-min “air breaks”), i.e., on five consecutive days followed by another session after 2 days of rest. At 30 min before the dive, and at 30 min, 2 and 4 h post-dive, exhaled breath was collected and followed by PFT. Exhaled breath samples were analyzed using gas chromatography-mass spectrometry (GC-MS). After univariate tests and correlation of retention times, ion fragments could be identified using a reference database. Using these fragments VOCs could be reconstructed, which were clustered using principal component analysis. These clusters were tested longitudinally with ANOVA.

**Results:**

After GC-MS analysis, eleven relevant VOCs were identified which could be clustered into two principal components (PC). PC1 consisted of VOCs associated with inflammation and showed no significant change over time. The intensities of PC2, consisting of methyl alkanes, showed a significant decrease (*p* = 0.001) after the first HBOT session to 50.8%, remained decreased during the subsequent days (mean 82%), and decreased even further after 2 days of rest to 58% (compared to baseline). PFT remained virtually unchanged.

**Discussion:**

Although similar VOCs were found when compared to diving, the decrease of methyl alkanes (PC2) is in contrast to the increase seen in divers. It is unknown why emission of methyl alkanes (which could originate from the phosphatidylcholine membrane in the alveoli) are reduced after HBOT. This suggests that HBOT might not be as damaging to the pulmonary tract as previously assumed. Future research on POT should focus on the identified VOCs (inflammation and methyl alkanes).

## Introduction

Hyperbaric oxygen therapy (HBOT) is increasingly used in the treatment of chronic (diabetic) wounds, post-radiation lesions and diving accidents (Lam et al., 2017; Mathieu et al., 2017; Zhao et al., 2017). Typically, HBOT administers oxygen at a partial pressure of 253 kPa (2.5 ATA) for a duration of 80–90 min for 30–60 daily sessions (Fosen and Thom, 2014; Stoekenbroek et al., 2015). HBOT oxygenates tissues and generates radical oxygen species (ROS). ROS act like a signaling molecule in pathways for a variety of growth factors, cytokines and hormones (Camporesi and Bosco, 2014; Fosen and Thom, 2014). After several sessions, HBOT promotes neovascularization, modulates the inflammatory system and promotes stem cells (Heyboer et al., 2014; Li et al., 2015; Tal et al., 2017). Besides the beneficial effects of HBOT, ROS can also lead to oxidative stress, possibly due to exhaustion or exceeding antioxidant capacity (Klein, 1990). Since patients receiving HBOT frequently report fatigue and pulmonary complaints after several sessions, HBOT is often paused in the weekends to allow patients to recover before the next week. Additionally, ROS are known to cause alveolar damage and inflammation to the respiratory system (van Ooij et al., 2016). However, other than generic symptoms like coughing or dyspnea, there are no objective or discriminating signs of clinical pulmonary oxygen toxicity (POT) (van Ooij et al., 2016; Wingelaar et al., 2017).

The discovery that oxygen is potentially damaging to the pulmonary system revealed the need for an upper limit of safe exposure (Smith, 1899). Early studies in this area included animal experiments and extreme exposures in human beings (Behnke et al., 1934; Bean, 1945). However, although insightful, these studies may never be replicated due to advances in research ethics. The studies of Clark and Lambertsen (in the late 1960s) laid the foundation for the Unit of Pulmonary Toxicity Dose (UPTD) (Bardin and Lambertsen, 1970; Clark and Lambertsen, 1970). In short: the UPTD quantifies oxygen exposure factoring in partial pressure of oxygen and time. In these latter experiments, where volunteers were exposed for 18 h to 100% oxygen at a pressure of 250 kPa, a correlation was found between decrease in vital capacity (VC) and time exposed to oxygen.

In diving, although, the UPTD concept was embraced worldwide, the original authors recognized several limitations. The most important one was the considerable intra- and interpersonal changes in VC, which were confirmed in later studies (Harabin et al., 1987). Additionally, according to the European Respiratory Society (ERS) Guidelines for PFT, VC is considered to be accurately measured when three measurements vary ≤0.15 L, which would be 2–3% of VC in adult males (Miller et al., 2005). Lastly, with a daily physiological variation in VC of 5%, it seems implausible that VC can be used to detect POT in a clinical setting, where a decrease in VC of 0.96% after a single HBOT session (equal to 144 UPTD) is expected (Arieli et al., 2002).

It was assumed that newer techniques, such as diffusion capacity, would be more accurate and affected to a lesser extent by these limitations. However, no valid parameter to quantify POT has been established (Wingelaar et al., 2017). Diffusion capacity, specifically the ratio between nitric oxide (DL_NO_) and carbon monoxide (DL_CO_), has been explored as a parameter to identify POT (van Ooij et al., 2014a). Although being able to distinguish between immersed dives with either air or 100% oxygen, this subtle change had little potential to quantify the amount of oxygen stress.

Exhaled breath analysis of volatile organic compounds (VOCs) after hyperbaric hyperoxia in divers has been reported in two studies (van Ooij et al., 2014b; Wingelaar et al., 2019). Generally, methyl alkanes were found. Several studies analyzed VOCs after normobaric hyperoxia and VOCs associated with oxidative stress and inflammation were found (Loiseaux-Meunier et al., 2001; Lemaitre et al., 2002; Phillips et al., 2003). No studies have investigated multiple dry hyperbaric hyperoxic exposures, such as HBOT. Therefore, we hypothesized that, after healthy volunteers received daily exposure to HBOT, the emission of methyl alkanes and VOCs associated with inflammation would increase due to cumulative pulmonary damage and normalize after 2 days of rest.

## Materials and Methods

### Setting

This prospective longitudinal cohort study was conducted at the Royal Netherlands Navy Diving Medical Center (Den Helder, Netherlands) and was carried out in accordance with the recommendations of the Ethics Committee of the University of Amsterdam. In accordance with the Declaration of Helsinki all participants gave written informed consent on a voluntary basis, which could be retracted at any time without any consequences. According to privacy regulations, no study data were included in the medical file of the participants. The protocol was approved by the Medical Ethical Committee of the University of Amsterdam (Reference: 2017.183) and the Surgeon General of the Ministry of Defence. The study was registered at the Dutch Trial Register (ID: NTR6547).

Eligible for inclusion were healthy, non-smoking personnel of the Royal Netherlands Navy, who were fit to dive according to the European Diving Technology Committee standards; with the exception that pulmonary function tests (PFT) were assessed using the reference values of the Global Lung Function Initiative (Wendling et al., 2004; Wingelaar et al., 2018). Exclusion criteria were: recent respiratory tract infection, daily use of two or more alcoholic beverages, and/or the use of (over-the-counter) medication.

Participants were not exposed to hyperbaric conditions for at least 72 h prior to start of the study. During the study and the day before hyperbaric exposure, no strenuous physical exercise (including sports) was performed. To avoid affecting the exhaled breath profile, divers had to fast for 1 h before the first measurement and were only allowed to drink water. Between the third and fourth measurement, food (bread and jelly) was provided and divers were encouraged to eat in order to prevent alteration of metabolism due to fasting (Fischer et al., 2015).

### Material and Measurements

Participants made daily “dry dives” of 95 min, including three 5 min “air breaks,” each to a pressure of 253 kPa (2.5 ATA) for 5 days (Monday to Friday) and the sixth dive after 2 days of rest (i.e., the following Monday). These HBOT sessions were performed in a Medusa treatment chamber (Haux Life Support, Germany). Participants breathed 100% oxygen via a breathing mask. No physical activity was performed at depth in order to standardize conditions and prevent central nervous system oxygen toxicity (Wingelaar et al., 2017).

Our procedures for PFT are published elsewhere; in short: spirometry, DL_NO_ and DL_CO_ were measured with a Masterscreen PFT Pro (Carefusion, Netherlands) by qualified respiratory technicians according to the ERS Guidelines (Miller et al., 2005; van Ooij et al., 2012; Graham et al., 2017). Baseline measurements were performed at least 24 h prior to or after participation in the study. To prevent forced expiratory maneuvers and exposure to carbon monoxide from affecting exhaled breath samples, PFT was performed daily after all exhaled breath samples had been collected (i.e., after collection of the fourth sample: see next paragraph).

Exhaled breath samples were collected as previously described (Wingelaar et al., 2019). The participant breathed for 5 min through a disposable two-way non-rebreathing valve (Carefusion, Netherlands) combined with an inspiratory VOC filter (Honeywell, United States) to prevent contamination of exogenous particles. After 5 min, a single expiratory breath was collected in an empty uncoated aluminum balloon (Globos Nordic, Denmark). After collection, 500 mL of exhaled breath was pumped through a stainless-steel tube filled with sorbent material (Tenax^TM^ GR 60/80, Camsco, United States) using a calibrated automatic air sampling pump (Gastec, Japan) at 250 mL/min, resulting in entrapment of VOCs. Pre-dive measurements were performed 30 min before hyperbaric exposure. Post-dive, the exhaled breath was collected at 30 min, 2 and 4 h.

Exhaled breath samples were analyzed using gas chromatography-mass spectrometry (GC-MS) based on standardized procedures (Horvath et al., 2017). In short, the tubes were heated to 250°C for 15 min with a flow of 30 mL/min using a thermal desorption unit (Markes, United States), where VOCs were captured in a cold trap at 10°C. Then, the cold trap was rapidly heated to 300°C for 1 min, after which molecules were splitless injected in a 30 m gas chromatography column with a diameter of 0.25 mm at 1.2 mL/min (Restek, United States). Molecules were ionized using electron ionization at 70 eV. Fragments were detected using a quadrupole mass spectrometer (GCMS-GP2010, Shimadzu, Japan) with a scan range of 37–300 Da. Ion fragments were used for statistical analysis. The predictive fragment ions were manually checked in the raw chromatograms and the corresponding metabolites were tentatively identified based on the National Institute of Standards and Technology (NIST) library matching, using the OpenChrom software package (Wenig and Odermatt, 2010). Metabolites were considered identified if the first five hits in the library were the same compound and all matching factors were above 90%.

### Statistical Analysis

Previous studies investigating VOCs after hyperoxia vary in methods of capturing, detecting and analyzing VOCs (Loiseaux-Meunier et al., 2001; Lemaitre et al., 2002; Phillips et al., 2003; van Ooij et al., 2014b; Wingelaar et al., 2019). As the techniques used affects the effect size, we can only refer to one study with similar capture and analysis techniques (Wingelaar et al., 2019). This latter study reported a 35% increase of emission of methyl alkanes after breathing 100% oxygen for 1 h at a pressure of 192.5 kPa. A similar effect was expected in the present study. Assuming a power of 80% and a significance level of 0.05 we needed a minimum sample of five participants to detect such an increase; however, to anticipate possible drop-out, we included ten participants.

After GC-MS analysis, an ion fragment peak table was generated, with de-noising, alignment and peak detection (signal-to-noise ratio 1:100) (Smith et al., 2006). A combined-batches algorithm was utilized to correct for possible batch effects (Johnson et al., 2007). Subsequently, data were tested univariately using Wilcoxon rank sum tests (i.e., pre- vs. post-dive or day 1 vs. day 2) to identify potentially relevant ion fragments. Then, ion fragments with retention times (±2 s) that correlated 0.98 or more were selected. From this selection of ion fragments/retention times, compounds could be identified. To analyze if compounds have a similar origin, a principal component (PC) analysis was performed. The means (intensity of the GC-MS signal) of the PCs were longitudinally tested using a two-way analysis of variance (ANOVA) with correction for participant and test day.

When assessing changes in PFT and diffusion over the course of the experiments, individual *t*-tests are likely to generate false-positive results. Due to the repeated nature of our measurements, a linear mixed model with each day as a covariate was considered appropriate to analyze longitudinal data (Cnaan et al., 1997). *Post hoc* analysis is not reliable in small test populations and was not performed.

All statistical analyses were performed using the R software package (version 3.5.1, R Foundation for Statistical Computing, Austria), including the surrogate variable analysis (SVA version 3.7), Methods for the Behavioral, Educational, and Social Sciences (MBESS version 4.4.3) and Combined Batches (ComBat version 3.28.0) packages. A *p*-value of <0.05 was considered statistically significant.

## Results

The study included ten healthy volunteers. During the trial, two female participants developed symptoms of upper respiratory tract infection and no longer met the inclusion criteria; therefore, these two participants were excluded from the analysis. An additional (male) participant could not attend on the last day of the study due to unexpected operational deployment. This individual was included in the analysis, but with missing data for the last study day. In total, eight volunteers completed the study. Baseline characteristics are presented in Table 1.

**Table 1:** Baseline characteristics of the study population.

	Total (*n* = 8)	Male (*n* = 5)	Female (*n* = 3)
Age (years)	35.8 (8.5)	33.3 (9.7)	40.5 (5.5)
Height (cm)	179.6 (4.6)	185.2 (3.7)	164.7 (4.5)
Weight (kg)	85.2 (8.8)	89.8 (10.7)	68.0 (8.2)
BMI	24.6 (3.1)	26.1 (2.5)	22.0 (2.4)

*NB, Data are after exclusion of two female participants who developed upper respiratory tract infection.*

### PFT Analysis

All parameters shown in Table 2 were individually tested in a linear mixed model. The values in the intercept column are the PFT values at baseline (before exposure). The coefficient from days 1–5 is the average change of the parameter per day over the first week. The coefficient at day six is shown separately to reveal the effect of “two days rest” between treatment days 5 and 6. None of the values were statistically significant.

**Table 2:** Linear mixed model parameters of PFT (*n* = 8).

	Intercept	Coefficient day 1–5	Coefficient day 6
FVC (L)	5.36 (5.01 – 5.72)	−0.04 (−0.25 – +0.19)	−0.11 (−0.66 – +0.44)
FEV_1_ (L)	4.11 (3.82 – 4.40)	−0.02 (−0.19 – +0.16)	−0.08 (−0.53 – +0.38)
DL_CO_ (mmol.min^−1^.kPa^−1^)	11.19 (10.06 – 12.32)	−0.11 (−0.54 – +0.33)	−0.39 (−2.14 – +1.36)
DL_NO_ (mmol.min^−1^.kPa^−1^)	43.91 (40.11 – 47.71)	−0.04 (−2.00 – +1.92)	−4.60 (−10.50 – +1.29)
DL_NO/CO_	4.09 (3.98 – 4.20)	+0.03 (−0.02 – +0.08)	−0.02 (−0.19 – +0.15)
VA_SB_ (L)	6.15 (5.79 – 6.52)	−0.01 (−0.24 – +0.23)	−0.12 (−0.68 – +0.45)
D_M_ (mmol.min^−1^.kPa^−1^)	22.40 (20.66 – 24.14)	−0.04 (−2.88 – +0.85)	−0.19 (−2.88 – +2.51)

### GC-MS Analysis

We planned to collect 240 GC-MS samples. However, as described, two participants were excluded and one could not attend the last test day. Additionally, due to logistic problems we were unable to collect data on the last two sampling moments (i.e., day six: 2 and 4 h post-dive). Also, 12 samples could not be analyzed for various reasons (e.g., too much noise, possibly due to contamination, or no signal possibly due to a faulty connection during sampling). Finally, 171 samples could be fully analyzed.

**FIGURE 1:**
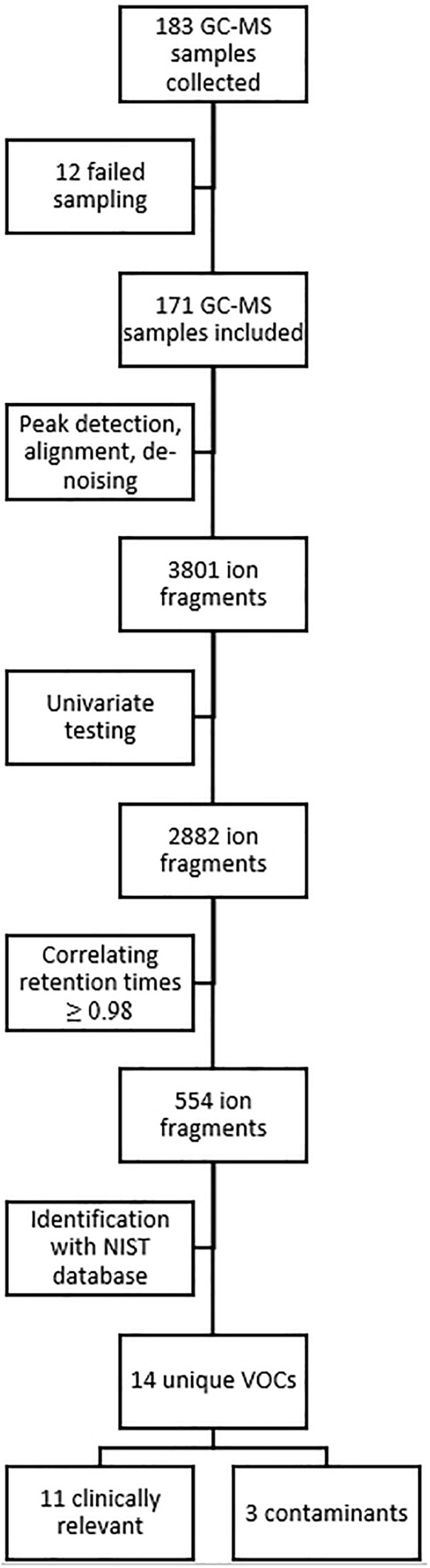
Overview of data and statistical analysis. GC-MS, gas chromatography-mass spectrometry; NIST, National Institute of Standards and Technology; VOC, volatile organic compound.

Analysis of these 171 samples led to the identification of 3801 unique ion fragments, of which 2882 were significant (*p* ≤ 0.05) in one or more instances when tested univariately (i.e., baseline vs. post-dive measurements, and measurements of day one vs. day two, etc). Of those 2882 ion fragments, 554 had a retention time (±2 s) that showed a correlation of ≥0.98. When grouping these fragments using the Standard Reference Database (NIST), 14 unique VOCs were identified (Figure 1).

**FIGURE 2:**
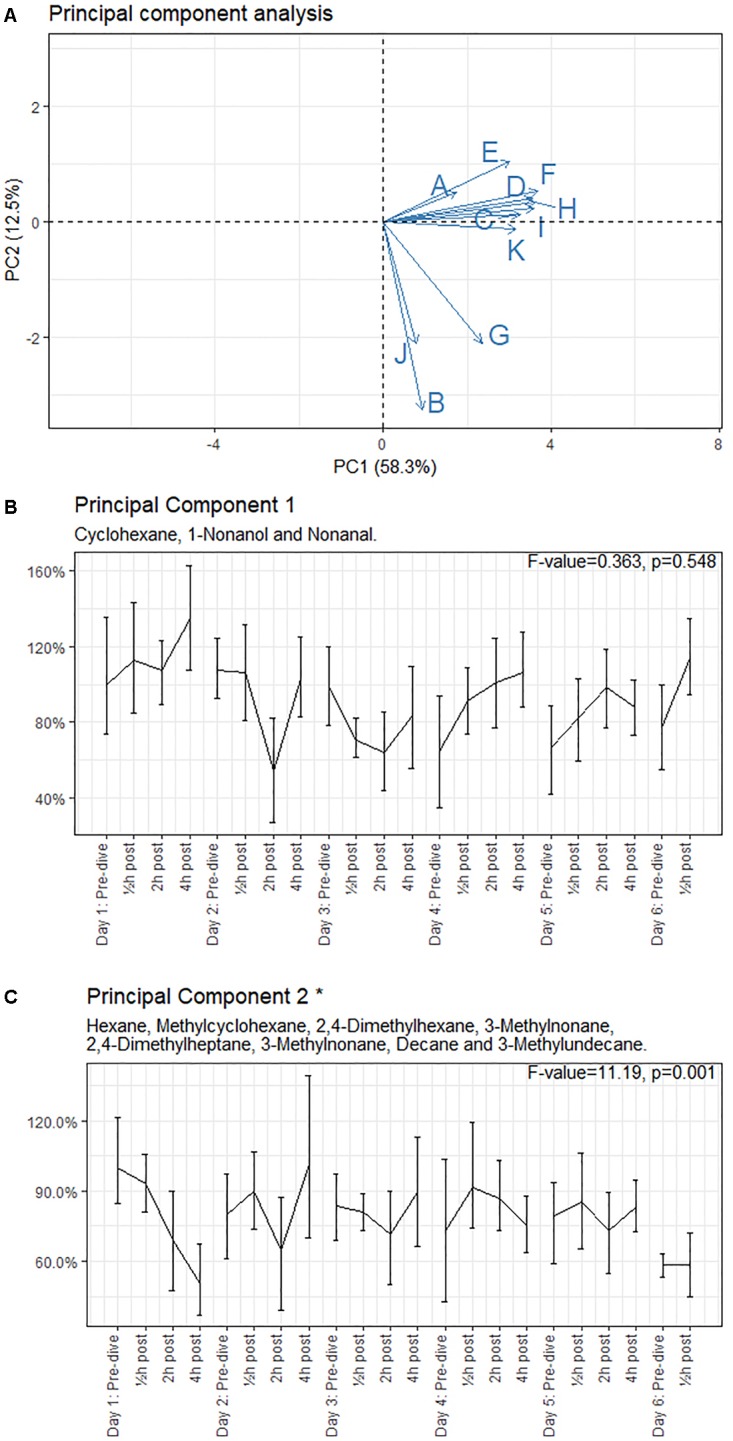
Principal component analysis loading plot and intensity of the principal components, with 95% CI. **(A)** Loading plot of the principal component (PC) analysis. The letters in panel **(A)** correspond to the compounds mentioned in Appendix 1. **(B,C)** show intensity, with 95% CI, of the PCs relative to the baseline measurement (day 1: pre-dive). ^∗^Significant difference in mean values. Results of the ANOVA are shown in the graph of each principal component.

Eleven compounds (generally methyl alkanes) were identified as being endogenous from origin and thus relevant for analysis; their intensity over time is reported in Appendix 1. The three remaining VOCs (Trisiloxane, Butyl Acetate and 1,1-Dichloropropane) are generally considered to be contaminants and were excluded from the analysis (D’ Angelo, 2011; National Center for Biotechnology Information, 2018). PC analysis showed that these 11 compounds could be clustered into two groups (Figure 2A). PC1 (Figure 2B) included cyclohexane, 1-nonanol and nonanal, and are associated with inflammation (Bos et al., 2014; Lamote et al., 2017; Chen et al., 2018). PC1 explained 58.3% of the variance. PC2 (Figure 2C) explained 12.5% of the variance and largely equalled the remaining identified components; these compounds could be related to damage to phosphatidylcholine (Phillips et al., 2003; Wingelaar et al., 2019).

The means of the PCs were tested using ANOVA (Figure 2B,C). The intensities of PC1 showed a non-significant (*p* = 0.548) increase in post-dive measurements on days 1, 4, 5, and 6, while the intensities of days 2 and 3 showed a decrease. The intensities of PC2 showed a significant decrease to 50.8% (CI 0.37–0.67, *p* = 0.001) on day 1 and remained at 65–101% (mean 82%) on the subsequent days, with a further decrease of intensity to 58% (CI 0.53–0.63) after the weekend (day 6).

## Discussion

After daily HBOT, 11 VOCs were identified which could be clustered into two PCs: markers of inflammation (PC1), and damage to phosphatidylcholine (PC2). PC1 showed no significant changes over time. PC2 was reduced ≥40% in the hours following the first HBOT, recovered to and remained around 80% for the subsequent days, and was further reduced to approximately 60% after 2 days of rest. All PFT parameters, including VC and diffusion capacity, remained almost unchanged. This confirms earlier studies suggesting that VOCs are a more sensitive marker than PFT to detect the onset of POT (van Ooij et al., 2014b; Wingelaar et al., 2019).

In the present study, the identified compounds are similar to those for hyperbaric hyperoxia in divers (van Ooij et al., 2014b; Wingelaar et al., 2019). In this study, the PC analysis, which separates the compounds into two groups, suggests a different biological origin of these two groups. Compounds in PC1 are commonly associated with pulmonary disease such as mesothelioma and ARDS in intubated ICU patients (Bos et al., 2014; Lamote et al., 2017; Chen et al., 2018). As we included only healthy subjects, these VOCs are likely to be caused by hyperbaric hyperoxia. The compounds in PC2 could originate from damage to the phosphatidylcholine membrane in the alveoli (Phillips et al., 2003; Chen et al., 2018; Wingelaar et al., 2019). This substantiates earlier hypotheses of oxygen affecting the alveoli, besides the inflammatory response (van Ooij et al., 2016).

In contrast to our hypothesis and results from our previous study in divers, the emission of methyl alkanes did not increase after daily hyperbaric hyperoxic exposure (Wingelaar et al., 2019). Moreover, the emission of methyl alkanes decreased after the first HBOT session. Furthermore, the intensity of the signal was significantly reduced to about 80% for days 2–5, and to about 60% after 2 days rest in the weekend. This suggests that there is (at least) no cumulative damaging effect of HBOT, and perhaps even adaptation to hyperbaric oxygen in a recompression chamber. Previous observations of upgraded radical scavengers in the type-II pneumocytes could be responsible for this process (Pietarinen, 2000). Several mechanisms could be responsible for these findings, none of which can be excluded with certainty from our experiments. Firstly, the PO_2_ is not continuous in HBOT. The “air breaks” (a 5-min pause of hyperbaric oxygen every 20 min, leaving the subjects breathing room air at 2.5 ATA with a PO_2_ of 0.5) could give the lung enough time to recover, similar to cerebral oxygen toxicity (Arieli et al., 2002). In rat models dry hyperbaric hyperoxic exposure of 90 minutes to 2.5 ATA did not induce changes in lung histology (Rubini et al., 2014). Secondly, it is known that immersion affects pulmonary function and cell physiology differently than dry hyperbaric hyperoxia. Not just by increased mechanical load due to immersion or decrease of pulmonary compliance due to higher gas densities, but also by immersion alone as shown *in vitro* models (Moon et al., 2009; Pendergast and Lundgren, 2009; van Ooij et al., 2011; Wang et al., 2015). Also, the water temperature could be an isolated confounder by increasing cellular energetic demands, possibly making them more susceptible to radical oxygen species (Pendergast and Lundgren, 2009). Lastly, the position of the diver (horizontal) versus the position of the subject in HBOT (vertical) affects transthoracic pressures, which affects breathing resistance (Moon et al., 2009). These factors, either singular or a combination of all of the above, could be responsible for the difference in emission of VOCs.

As in our previous study using exhaled breath analysis in divers, an interval of 2–4 h after hyperbaric hyperoxic exposure and collection of breath samples gives better results than measuring directly after exposure (Wingelaar et al., 2019). This is in line with previous studies (Loiseaux-Meunier et al., 2001; Lemaitre et al., 2002).

Even though the results of the present study strongly indicate the value of further investigating POT using GC-MS instead of PFT, some items need to be addressed. Firstly, the reference values of “normal” are unknown (Pereira et al., 2015). We have presented our results as a relative increase or decrease from baseline. This is sufficient for scientific purposes, but difficult for clinical implementation to evaluate whether an individual patient is suffering from POT. GC-MS analysis is a time and resource consuming technique which requires highly trained personnel. A possible direction for future research is to use “electronic noses” (eNose) for exhaled breath analysis (Fens et al., 2013). These devices are increasingly stable and show promising results in the field of pulmonary medicine (Brinkman et al., 2018). Since the measurements are non-invasive, easy to perform (after a short instruction), relatively cheap and the results can be directly available (point of care), this seems an ideal method for the collection of large amounts of samples. Whether “electronic nose” technology can detect markers of inflammation and exhaled methyl alkanes remains to be investigated.

Three identified compounds are generally considered to be contamination: Trisiloxane, 1,2-Dichloropropane and butyl acetate. The first is commonly found in GC-MS analysis and the results of column or septum bleed (D’ Angelo, 2011). Silicon is not a part of human physiology and was therefore excluded from analysis. 1,2-Dichloropropane could originate from the breathing masks used to supply the breathing gas. Lastly, butyl acetate was identified at several post-dive measurements, overlapping with consumed meals. However, butyl acetate is commonly used as a flavoring agent and probably originates from the diet we gave our participants (bread and jelly) (National Center for Biotechnology Information, 2018). Although this influenced our data, total fasting for 7 h would probably have had more impact on our results.

## Strengths and Limitations

This is first the study to longitudinally collect VOCs after daily hyperbaric hyperoxic exposure in healthy volunteers. The multi-day exposure provides clinical relevance to hyperbaric oxygen treatment. The use of healthy volunteers excluded potential confounders from pathology in patients undergoing HBOT. Also, this study included both males and females, thereby increasing the practical relevance.

Some limitations need to be addressed. First, this study has included a small number of subjects, was unblinded and lacked a control group. However, to create a valid control group for HBOT presents ethical and practical difficulties (Lansdorp and van Hulst, 2018). Whether an unblinded inclusion affected the results of GC-MS or PFT is unknown, but seems unlikely. As mentioned, the reference values of many of these VOCs are unknown (Pereira et al., 2015). However, since we included participants early in the morning and late in the afternoon, at least some of the potential circadian variation should have been balanced out. Also, as our results are in line with different study populations, collection methods and sample techniques, this tends to confirm that our results are not subject to selection bias or natural variation. Although male and female physiology could respond differently to hyperbaric hyperoxic conditions, we choose to include both sexes in this study to increase its clinical relevance (van Ooij et al., 2011; Lautridou et al., 2017). However, in a large study by Blanchet et al. (2017) no significant effect of age, sex or BMI on exhaled breath profiles was found. Even though the groups are too small to perform a reliable subgroup analysis, a similar response was seen in both groups. As our study only included healthy individuals, further studies are required to evaluate whether a similar breath profile can be detected in clinical patients.

Second, similar studies often include systemic biomarkers of oxidative stress, such as malondialdehyde in blood or subfractions of hydroxybenzoate in urine (Loiseaux-Meunier et al., 2001; Gronow et al., 2005). However, systemic markers might originate from organ systems other than the lung and are, therefore, not entirely specific (Frijhoff et al., 2015; Valacchi et al., 2018). We think that our results are organ specific and generate sufficient evidence for oxidative damage of alveolar membranes and inflammation.

Lastly, due to the high number of samples, the GC-MS analysis was performed in several batches. This could introduce systemic bias, potentially masking effects or introducing false-positive results. To avoid this, we analyzed the GC-MS samples in random order and not in the order in which they were collected. Any batch effects were further reduced by applying Combined Batch correction. Arguably the strongest argument to conclude that our results are not a result of systemic bias, is the different vector from PC1 and PC2. If these effects had been the result of batch effects, their vector would overlap. Additionally, the methods of collection, identification and analysis of VOCs differed from earlier studies, and similar VOCs (methyl alkanes) were identified after hyperbaric hyperoxic exposure (van Ooij et al., 2014b). We feel this supports the idea that our results are not the result of systemic or selection bias.

## Conclusion

In this study, after daily HBOT for 6 days, we identified 11 VOCs which could be divided into two PCs. PC1 (inflammation) showed no significant changes over time, PC2 (methyl alkanes) was significantly reduced after daily exposure for 5 days, to approximately 60% after 2 days’ rest. This suggests that daily HBOT does not induce cumulative damage to the pulmonary system of healthy volunteers, but triggers an adaptive response. Additional studies including more treatment sessions, and perhaps additional systemic parameters on radical oxygen scavengers and inflammatory markers, are necessary to substantiate this finding. To evaluate clinical application of these findings, studies should explore the use of “electronic nose” technology as a surrogate for GC-MS and, preferably, include patients.

## Data Availability

The datasets generated for this study are available on request to the corresponding author.

## Ethics Statement

This prospective longitudinal cohort study was conducted at the Royal Netherlands Navy Diving Medical Center (Den Helder, Netherlands) and was carried out in accordance with the recommendations of the Ethics Committee of the University of Amsterdam. In accordance with the Declaration of Helsinki all participants gave written informed consent on a voluntary basis, which could be retracted at any time without any consequences. According to privacy regulations, no study data were included in the medical file of the participants. The protocol was approved by the Medical Ethical Committee of the University of Amsterdam (Reference: 2017.183) and the Surgeon General of the Ministry of Defence. The study was registered at the Dutch Trial Register (ID: NTR6547).

## Author Contributions

TW conceived the idea, designed and performed the experiments, carried out the statistical analysis, and drafted and revised the manuscript. PvO and RvH conceived the idea, designed the experiments, and drafted and revised the manuscript. PB designed and performed the experiments, carried out the GC-MS and statistical analysis, and drafted and revised the manuscript. RH, AM-vdZ, and MH conceived the idea, designed the experiments, and revised the manuscript.

## Conflict of Interest Statement

The authors declare that the research was conducted in the absence of any commercial or financial relationships that could be construed as a potential conflict of interest.

## Supplementary Material

APPENDIX 1 Intensity of the VOCs, with 95% CI. ^∗^Significant difference in mean values. Results of the ANOVA are shown in the graph of each VOC.
