# Supplementary figures and images for: Markers of Pulmonary Oxygen Toxicity in Hyperbaric Oxygen Therapy Using Exhaled Breath Analysis
Source: Front Physiol. 2019 Apr 24;10:475. doi: 10.3389/fphys.2019.00475 (PMC6491850; doi:10.3389/fphys.2019.00475)

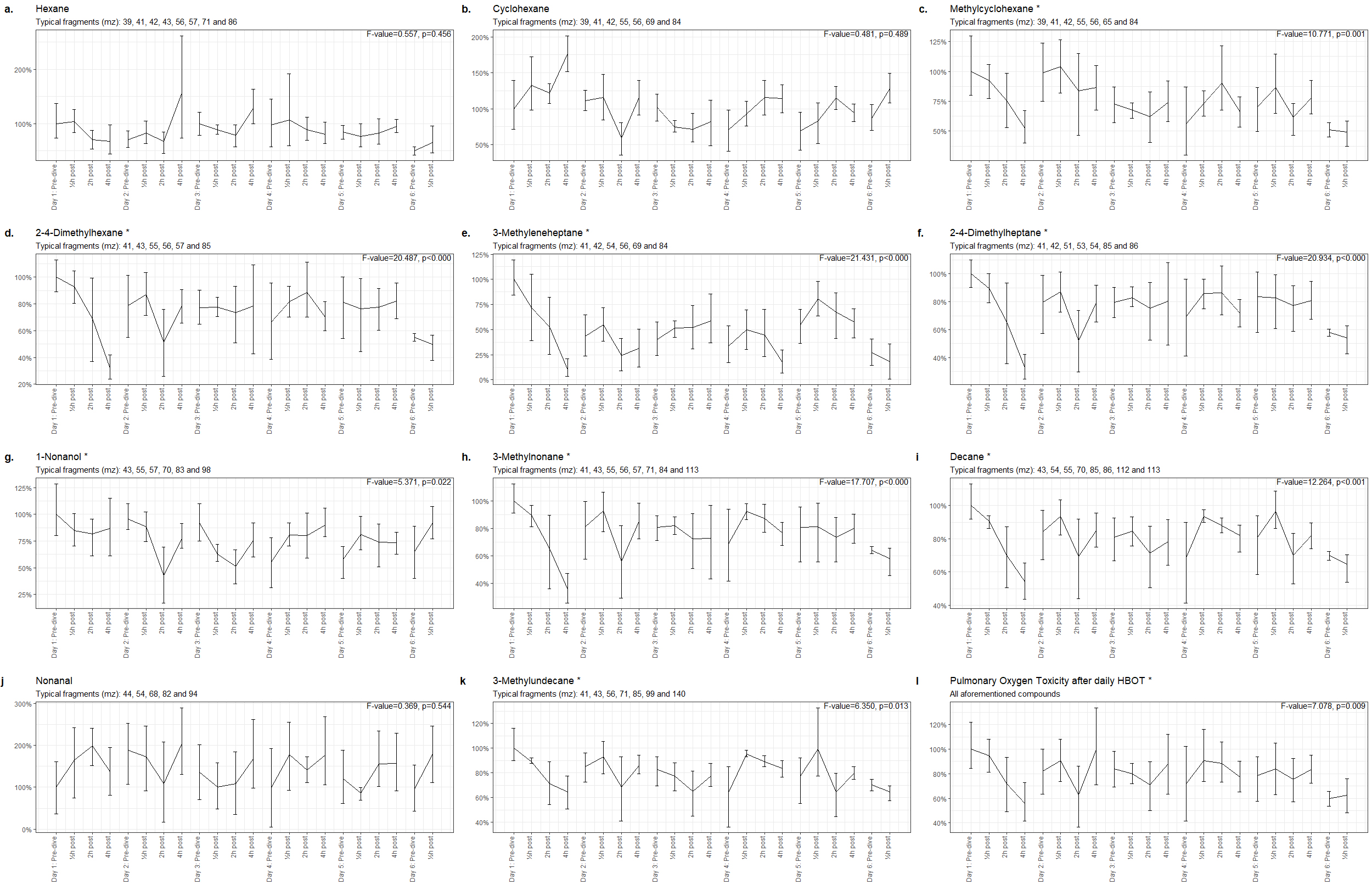

Supplement: APPENDIX 1 — Intensity of the VOCs, with 95% CI. ∗Significant difference in mean values. Results of the ANOVA are shown in the graph of each VOC. [file Image_1.JPEG]
